# Removal of Total Coliforms, Thermotolerant Coliforms, and Helminth Eggs in Swine Production Wastewater Treated in Anaerobic and Aerobic Reactors

**DOI:** 10.1155/2014/757934

**Published:** 2014-04-09

**Authors:** Silvia Helena Zacarias Sylvestre, Estevam Guilherme Lux Hoppe, Roberto Alves de Oliveira

**Affiliations:** Programa de Pós-Graduação em Microbiologia Agropecuária, Faculdade Ciências Agrárias e Veterinárias, Universidade Estadual Paulista “Júlio de Mesquita Filho” UNESP, Via de Acesso Prof. Paulo Donato Castellane s/n, 14884-900 Jaboticabal, SP, Brazil

## Abstract

The present work evaluated the performance of two treatment systems in reducing indicators of biological contamination in swine production wastewater. System I consisted of two upflow anaerobic sludge blanket (UASB) reactors, with 510 and 209 L in volume, being serially arranged. System II consisted of a UASB reactor, anaerobic filter, trickling filter, and decanter, being also organized in series, with volumes of 300, 190, 250, and 150 L, respectively. Hydraulic retention times (HRT) applied in the first UASB reactors were 40, 30, 20, and 11 h in systems I and II. The average removal efficiencies of total and thermotolerant coliforms in system I were 92.92% to 99.50% and 94.29% to 99.56%, respectively, and increased in system II to 99.45% to 99.91% and 99.52% to 99.93%, respectively. Average removal rates of helminth eggs in system I were 96.44% to 99.11%, reaching 100% as in system II. In reactor sludge, the counts of total and thermotolerant coliforms ranged between 10^5^ and 10^9^ MPN (100 mL)^−1^, while helminth eggs ranged from 0.86 to 9.27 eggs g^−1^ TS.

## 1. Introduction

Pig farming has greatly intensified in recent years. Total swine numbers in 2011 reached 39.3 million units in Brazil, up 0.9% from 2010 according to the Brazilian Institute of Geography and Statistics (IBGE) [1].

The intensification of feedlot swine production is responsible for producing large amounts of liquid waste, which once released without treatment into nature can pollute water springs, affect air quality from gas emissions, and cause insect proliferation [2].

Feedlot conditions result in high prevalence of pathogenic microorganisms on floor surfaces, as the digestive and urinary systems of pigs are their main routes of waste disposal. It is important to take into account that waste allows pathogen survival and dissemination for days to months [3].

The public health aspect, as one of the most relevant aspects of using effluents for productive purposes, is still object of controversy in the international technical-scientific community. There are still controversies with regard to admissible risks, and by extension, the necessary and sufficient quality of effluents in order to guarantee health protection. The consensus extends only to the acknowledgment that irrigation using untreated wastewater offers real risks of transmitting diseases, and that any irrigation practice using sewage involves public health risks. Nevertheless, there are still controversies with regard to admissible risk levels, and by extension, the level of treatment and the necessary and sufficient quality of effluents in order to guarantee health safety [4–6].

In Brazil, there are no specific rules establishing parasitological parameters for the reuse of low-quality water. Therefore, the guidelines set by the World Health Organization are followed [7, 8].

For microbiological parameters, resolutions 357/2005 and 375/2006 by Brazil's National Environmental Council (CONAMA) are noteworthy. CONAMA resolution 357/2005 establishes standards for water quality and uses and consequently for effluent release in water bodies. And CONAMA resolution 375/2006 deals specifically with sewage sludge. There is no specific legislation for farming waste or similar [9, 10].

CONAMA resolution 375 from 2006 uses microbiological and parasitological parameters to classify sludge for use as fertilizer in agriculture, into type A (or derived product) or type B. Sludge is classified as type A when the concentration of thermotolerant coliforms is below 10^3^ MPN g^−1^ TS, and viable helminth eggs are below 0.25 egg g^−1^ TS. For sludge to be classified as type B, the concentration of thermotolerant coliforms must be over 10^3^ and below 10^6^ MPN g^−1^ TS, and helminth eggs should be over 0.25 g^−1^ TS and below 10 eggs g^−1^ TS [10].

According to Van Haandel and Marais [11], the guidelines recommended by the World Health Organization are based on theoretical models, epidemiological evidence, and information available on the efficiency of pathogen removal, particularly through the use of stabilization ponds.

The bacteriological and parasitological standards recommended by the WHO forum restricted irrigation are 10^3^ MPN (100 mL)^−1^ and 1 helminth egg L^−1^, respectively. American standards require the absence of pathogenic indicators (including viruses and protozoa) for unrestricted irrigation [5, 12].

Therefore, to use the treated effluent for irrigation and/or fertilization and reactor sludge fed with wastewater, it is essential to know its physical-chemical characteristics and microbiological contamination indicators, in order to establish adequate environmental protection measures and the appropriate choice of technologies for treatment and final disposal.

The use of anaerobic reactors as a secondary treatment contributes to remove pathogens. Moreover, according to Chernicharo [13], some of the advantages of anaerobic treatment are low energy consumption, very low operational costs, small space requirements, methane production, and tolerance against high organic loads. Among anaerobic treatment systems, the upflow anaerobic sludge blanket (UASB) reactor stands out. In the UASB reactor, a significant portion of suspended solids present in raw sewage (including helminth eggs) are retained, which are retained in the bed of thick biological sludge given their density and due to hydraulic upflow. In addition to this retention of solids in the bottom of the reactor, there is also sedimentation of biological sludge that eventually seeps out from the sludge digestion compartment, but this requires installation of a solids separator in the upper part of the tank.

Despite the advantages attributed to anaerobic reactors, the effluent produced usually does not meet quality standards set by environmental legislation, with regard to the number of helminth eggs, total, and thermotolerant coliforms, thus requiring the addition of a posttreatment system.

One alternative for posttreatment is the trickling filter (TF), which works with continuous feeding and without unit flooding. It is an aerobic reactor, permanently subject to air replacement, which naturally circulates in the empty spaces of the support medium, providing the necessary oxygen for microorganism respiration [14].

According to Van Haandel and Marais [11], the combined anaerobic/aerobic system has great potential in reducing construction and operational costs. Several works have been carried out using this combination to treat swine production wastewater, aiming to remove coliforms, among others. Duda and Oliveira [15] used a system consisting of anaerobic reactors (UASB and anaerobic filter) and aerobic reactors (trickling filter). Oliveira and Santana [16] used two UASB reactors followed by an aerobic sequencing batch reactor (SBR), and Santos [14], who also worked with an anaerobic/aerobic/anoxic treatment system, obtained significant coliform removal efficiency.

This work evaluated two treatment systems: one with two UASB reactors in series and another consisting of UASB reactors, anaerobic filter, trickling filter, and decanter, placed in series, in the removal of coliforms and helminth eggs from swine production wastewater.

## 2. Materials and Methods

**Figure 1:**
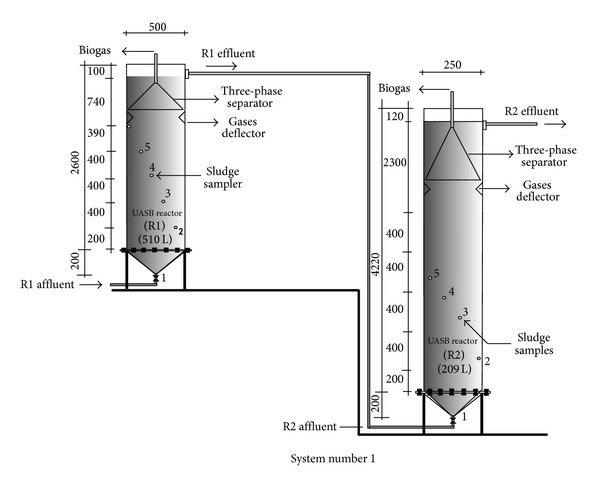
Schematic diagram of the experimental facilities of anaerobic treatment system I (I), with two UASB reactors, placed in series, at pilot scale. Source: Oliveira and Santana [16].

The experimental facilities consisted of two treatment systems. System I consisted of two UASB reactors, with volumes of 510 and 209 L, placed in series (Figure 1), as described by Oliveira and Santana [16].

**Figure 2:**
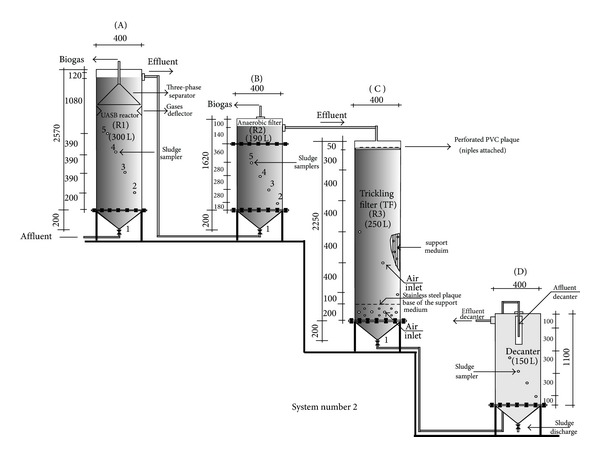
Experimental facility design for treatment system II consisting of UASB reactor, upflow anaerobic filter, trickling filter, and decanter placed in series, at pilot scale. Source: Duda and Oliveira [15].

System II consisted of a UASB reactor, anaerobic filter, trickling filter, and decanter, placed in series, with volumes of 300, 190, 250, 150 L, respectively (Figure 2), as described by Duda and Oliveira [15]. In the anaerobic and aerobic biological filters, polypropylene rings were used as support medium, with specific surface area of 101 m^2^/m^3^.

The swine production wastewaters utilized to feed the treatment systems were collected daily in a feedlot for growing and finishing swine, at a commercial property located in the city of Jaboticabal, SP, which uses shallow water channels to transport the waste. The collected wastewater was first sieved (3 mm mesh) to separate rough solids; next, the water was stored in boxes and pumped to the first-stage reactors. The other reactors were gravity fed. The operating conditions applied on the systems are described in Table 1.

### 2.1. System Monitoring

Monitoring of the reactors began in July 2011 and lasted until September 2012. Collections were carried out every fortnight following stabilization of treatment systems in each assay.

The anaerobic reactors were regarded as stable when the coefficient of variation (CV) values of the removal efficiencies of total COD and volatile suspended solids (VSS), concentration of volatile acids (TVA), and methane production (CH_4_) were lower than 20%; in the trickling filter, whenever average COD and VSS values in the effluent and their efficiencies had CV below 20%.

Inflow and outflow samplings were carried out at the end of the assays, after 60 days of operation in assay 1, 30 days in assay 2, 60 days in assay 3, and 75 days in assay 4. The sludge was collected in the same period.

The laboratory exams performed were total and thermotolerant coliform counts and number of helminth egg in inflows, effluents, and reactor sludge and decanter.

### 2.2. Determination of Total and Thermotolerant Coliforms

To determine coliforms, the multiple-tube technique was used in accordance with CETESB norm L.5 202 [17] and in conformity with the Standard Methods for the Examination of Water and Wastewater. The results were expressed in MPN (most probable number) per 100 mL of sample [18].

The samples were collected in autoclaved glass flasks and processed immediately after collection.

Samples of the inflows were collected in the incoming pipes of the anaerobic reactors and from outflows in the outgoing pipes of the anaerobic reactors, trickling filter, and decanter. The collections were performed at the end of each assay, with two replications per assay. Sludge samples were collected at all sampling points.

### 2.3. Determination of Helminth Eggs

For processing, sample preparation, and counting of helminth eggs, the study used the sedimentation method developed by Bailenger [19] and modified by Ayres and Mara [20]. This method was chosen due to its simplicity and the low cost of the reagents used, in addition to the fact that it allows recovery of a wide range of helminths usually found in wastewaters, particularly nematode eggs (*Ascaris *sp.,* Trichuris *sp., and hookworms) which are the specific parasitological indicators found in the World Health Organization guide for reuse in agriculture [21].

The samples from inflows and outflows (10 L of each) were collected and placed in 15 L polyethylene drums and processed after two hours of sedimentation. The sampling site for the inflows was in the incoming piping of the anaerobic reactors. Outflows were sampled in the outgoing pipes of the anaerobic reactors, trickling filter, and decanter. Two replications were carried out per assay.

The Meyer method was used to recover helminth eggs from the sludge [22]. The results were expressed as eggs g^−1^ TS [23].

The sludge samples were collected at two collection points along the reaction chamber of the reactors: (1) UASB reactors in system I, sludge bed and blanket, at points 400 and 1200 mm from the inflow entrance (Figure 1); (2) UASB reactor in system II, sludge bed and blanket, at points 400 and 1180 mm from the inflow entrance; (3) anaerobic filter in system II, at points 380 and 940 mm from the inflow entrance; (4) decanter in system II, 1100 mm from the inflow entrance (Figure 2). At each point, a 1-L sample of sludge was collected and placed in polyethylene bottles.

## 3. Results and Discussion

### 3.1. Total and Thermotolerant Coliforms in Inflows and Effluents

The mean values of the numbers of total and thermotolerant coliforms in the inflows of systems I and II ranged between 1.40*E* + 07 and 2.40*E* + 08 MPN (100 mL)^−1^ in assays 1 to 4. In the effluents of UASB reactors (R1), they decreased to mean values of 1.50*E* + 06 to 4.40*E* + 07 MPN (100 mL)^−1^ as shown in Tables 2 and 3.

In the effluent of the UASB reactor (R2) of treatment system I, the reduction was maintained to mean values of total and thermotolerant coliforms of 1.90*E* + 05 to 1.70*E* + 07 MPN (100 mL)^−1^ in assays 1 to 4 (Table 2). In the anaerobic filter of treatment system II there were decreases as well, down to mean values of 4.30*E* + 05 to 2.00*E* + 07 MPN (100 mL)^−1^ of total and thermotolerant coliforms, in assays 1 to 4 (Table 3). With the addition of TF, a decrease was obtained to values of 2.00*E* + 04 to 2.20*E* + 06 for assays 1 to 4. In the decanter, the reduction was maintained to mean values of total and thermotolerant coliforms of 2.40*E* + 04 to 1.20*E* + 06 in assays 1 to 4. In treatment system I, with two UASB reactors in series, an effluent with superior microbiological quality for the evaluated coliform indicators, except in assay 4, when the values were identical for total coliforms. In both treatment systems, in the anaerobic reactors, the effect of HRT reduction became evident, especially in assay 4, hindering the microbiological quality of the effluent by raising the count of total and thermotolerant coliforms.

Neto [24] obtained higher values of total thermotolerant coliforms in swine production wastewater of 1.00*E* + 07 MPN (100 mL)^−1^ similar values as those found in the present work.

Santos et al. [25] obtained higher values of total coliforms, totaling 1,00*E* + 08 to 1,00*E* + 10 MPN (100 mL)^−1^ and thermotolerant coliforms values of 1,00*E* + 08 to 1,00*E* + 09 MPN (100 mL)^−1^.

Oliveira and Santana [16] obtained similar or slightly higher results when evaluating coliform concentrations in swine production wastewater. After treatment in both UASB reactors of system I, the authors also obtained a marked reduction in the concentrations of thermotolerant coliforms in the effluent of R1 to average values of 4.30*E* + 06 to 4.30*E* + 07 MPN (100 mL)^−1^ and of R2, which decreased to average values of 2.40*E* + 06 to 4.30*E* + 07 MPN (100 mL)^−1^.

Therefore, the evaluated anaerobic treatment systems revealed a reduction potential of two logarithmic units for the removal of total and thermotolerant coliforms, with relatively small variations among assays 1 to 3, in which the HRT was 59 to 28 h. With the decrease of HRT to the range of 15 to 16 h in assay 4, the removals were in the order of one logarithm.

**Figure 3:**
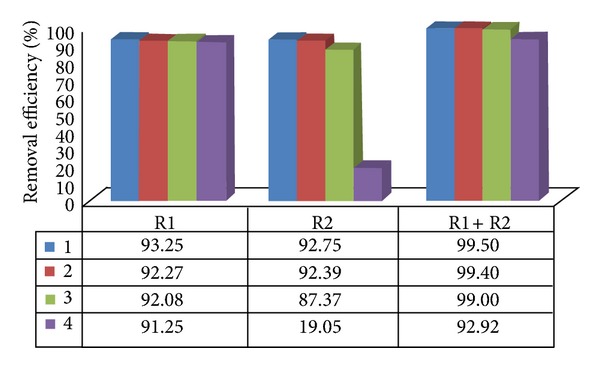
Removal efficiency of total coliforms in the UASB reactors (R1 and R2) placed in series, at pilot scale, for treatment system I, in assays 1, 2, 3, and 4.

**Figure 4:**
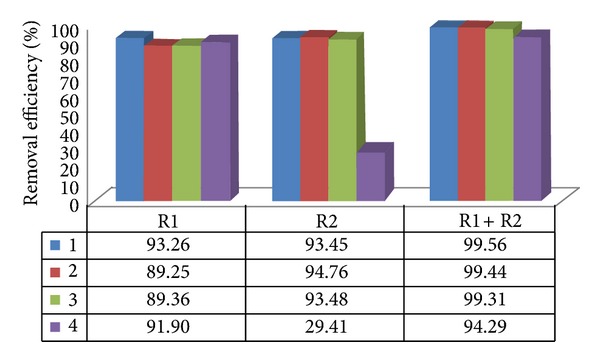
Removal efficiency of thermotolerant coliforms in the UASB reactors (R1 and R2) placed in series, at pilot scale, for treatment system I, in assays 1, 2, 3, and 4.

**Figure 5:**
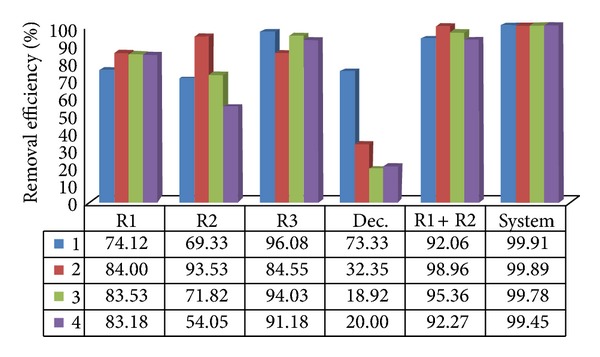
Removal efficiency of total coliforms in treatment system II, consisting of UASB reactor, anaerobic filter (AF), trickling filter (TF), and decanter (D) placed in series, in assays 1, 2, 3, and 4.

**Figure 6:**
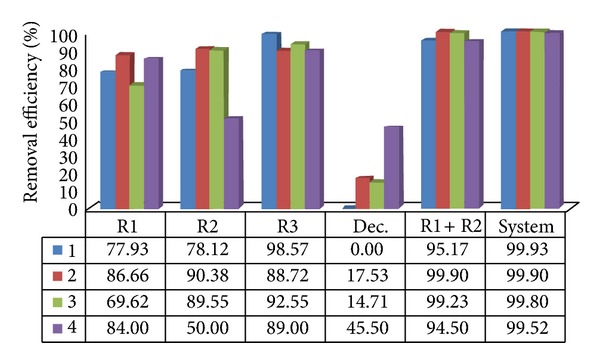
Removal efficiency of thermotolerant coliforms in treatment system II, consisting of UASB reactor, anaerobic filter (AF), trickling filter (TF) and decanter (D) placed in series, in assays 1, 2, 3, and 4.

The average removal efficiencies of total and thermotolerant coliforms were 92.92% to 99.93% in the anaerobic reactors of treatment systems I and II in assays 1 to 4 (Figures 3, 4, 5, and 6). The highest efficiencies for removal of coliforms occurred in assays 1, 2, and 3. The lower HRT and temperature in assay 4 resulted in lower coliform removal and were caused principally by the reduced efficiency in the UASB reactor (R2) and anaerobic filter of treatment systems I and II, in which the HRTs were 4.5 and 5.2 h, respectively.

Even while achieving 99.93% removal efficiency of thermotolerant coliforms in the effluents of anaerobic reactors of treatment systems I and II, the coliform concentrations still exceeded standards established for use in plant irrigation according to Brazilian legislation CONAMA 357/2005 and for treated outflows according to the guidelines set by the World Health Organization [7, 8].

Analyzing swine production wastewater from UASB reactor and anaerobic filter with HRT 12.0 and of 8.5 h, respectively, Pereira-Ramirez et al. [26] obtained concentrations of thermotolerant coliforms in the final effluent of 2.51*E* + 07 MPN (100 mL)^−1^. The anaerobic filter (AF) is removed between 80 and 96% of thermotolerant coliforms, similar and higher values than those obtained in this work.

Buzato [27], assessing the performance of an upflow anaerobic filter in the treatment of domestic sewage using a UASB reactor, obtained an average removal efficiency of total and thermotolerant coliforms of 81 and 78%, respectively. Average removal efficiency in the UASB reactor was 71% for total coliforms and 69% for thermotolerant coliforms. The anaerobic filter showed average removal efficiencies of total and thermotolerant coliforms of 41% and 33%, respectively, lower values than that found in this work.

Duda [28] worked with system II in the treatment of swine production wastewater; the highest efficiencies obtained in the removal of thermotolerant coliforms were 99.86% in the UASB reactor and anaerobic filter by themselves. After adding the trickling filter (TF) as posttreatment, the efficiencies were 99.94 and 99.99% in the assays with HRT of 44.7 and 66.6 h for the system of anaerobic treatment and posttreatment higher values than that obtained in this work, which demonstrates that greater HRT results in more significant removal efficiency.

System II resulted in higher removal efficiency values than system I, consisting only of the UASB anaerobic reactors in series, confirming the importance of posttreatment, as shown in Figures 5 and 6.

The adequacy of bacteriological quality in the final effluent, in order to meet the standards of environmental legislation, will depend essentially on the characteristics of the receiving water body. In that sense, in order to meet quality standards of a class 2 river at most 1.00*E* + 03 MPN (100 mL)^−1^, the dilution and concentration of coliforms upstream from the discharge points will be preponderant factors in the analysis [9, 15, 16, 28].

### 3.2. Total and Thermotolerant Coliforms in Sludge

In UASB (R1) and UASB (R2) reactors of system I, a higher concentration of coliforms was observed in the bed of biological sludge, point 1, which is located at the bottom of the reactor. The average concentrations of total coliforms at point 2 were 1.50*E* + 08 to 9.10*E* + 08 MPN (100 mL)^−1^ and thermotolerant coliforms concentrations ranged between 1.10*E* + 08 and 6.50*E* + 08 MPN (100 mL)^−1^ in all assays.

At the points 4 and 5 (Figure 1), in the biological sludge blanket, which is located at the top of the reaction chamber from the UASB reactor (R1), the concentrations of coliforms decreased one or two logarithmic units, as shown in Table 4. These numbers of coliforms remained in the bed of biological sludge from the second UASB reactor (R2), in which it also there was reduction of one more logarithmic unit in the sludge blanket.

With regard to the sludge in system II, at point 1 of the UASB reactor and AF, the concentration of coliforms was also higher compared to the other points in all assays and decreased as the distance from the reactor bed increased, as shown in Table 5.

In the treatment of domestic sewage using UASB reactors, Backes [29] evaluated sludge and obtained thermotolerant coliform values of 2.10*E* + 03 MPN g^−1^ TS, classifying it as type-B sludge, giving the possibility of reuse, similar to the values found in this work. Santos et al. [25], working at the Sewage Treatment Plant of Rios das Antas, using UASB reactors, operated by the Paraná State Sanitation Utility, found concentrations of thermotolerant coliforms below 10 MPN g^−1^TS; as such, that residue could be used as fertilizer in crop soils.

It is worth reminding that there is no specific legislation for sludge from the swine treatment wastewaters. CONAMA Resolution 375 [10] defines criteria and procedures for the agricultural use of sewage sludge created in sewage treatment plants and their byproducts.

To reuse sludge in agriculture (type A), the concentrations of total and thermotolerant coliforms must stay below 1.00*E* + 03 MPN g^−1^ TS and below 1.00*E* + 06 MPN g^−1^ TS for type B. Only sludge produced in the UASB reactor (R2) of system I (assays 1, 2, 3, and 4) showed values that are in accordance with the standards set by CONAMA Resolution 375 [10] for reuse in agriculture as type-B sludge.

*Parasitological Analysis*

### 3.3. Determination of Helminth Eggs in Inflows and Effluents

Table 6 shows the results of the identification and count of the average number of helminth eggs obtained in the samples of inflow and outflow of UASB reactors (R1 and R2) placed in series, at pilot scale, of treatment system I, in assays 1, 2, 3 and 4. Only eggs of* Ascaris suum *were found.

Morris et al. [30], while comparing swine farms in slotted or cement floors, described greater occurrence of* Ascaris suum *on cement. Facilities with shallow pools are conducive to the dissemination of parasite agents when inadequately used, which explains the presence of* Ascaris suum *eggs in the inflow. It is important to assess water flow, so that waste is constantly discharged from the area.

The concentration of parasite forms in swine production wastewater is associated with handling practices and facilities. Changes in the raising system reduce infection rates, but the agents can persist even in properties with good handling practices [31]. The use of ivermectin injections in pregnant sows prevents vertical transmission from sows to piglets and consequently reduce local contamination. Treatment with anthelminthics is done using oral fenbendazole, and all animals are kept in a cemented area without access to soul, receiving only commercial feed. Therefore, helminth resistance in herds is associated with contamination and environment resistance by the eggs of these helminths.

**Figure 7:**
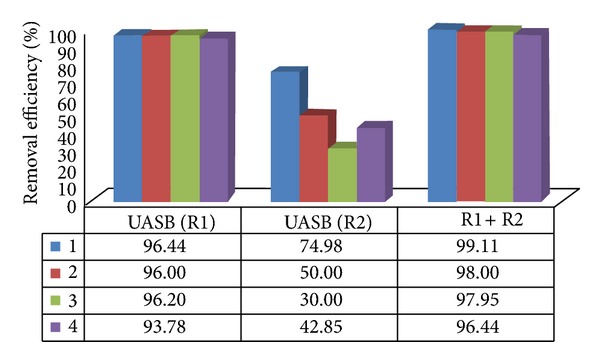
Average values of the removal efficiencies of* Ascari ssuum* eggs in UASB reactors (R1 and R2) and R1 + R2 of treatment system I in assays 1, 2, 3, and 4.

In the inflow, the number of* Ascaris suum *eggs found in system I ranged from 2.3 to 3.0 eggs L^−1^, averaging 2.5 eggs L^−1^ overall for system I. In the effluent of the two stage UASB reactors of system I, lower counts were obtained, averaging 0.027 to 0.107 eggs L^−1^, with removal efficiency between 96.44 and 99.11% in assays 1 to 4, as shown in Figure 7.

**Figure 8:**
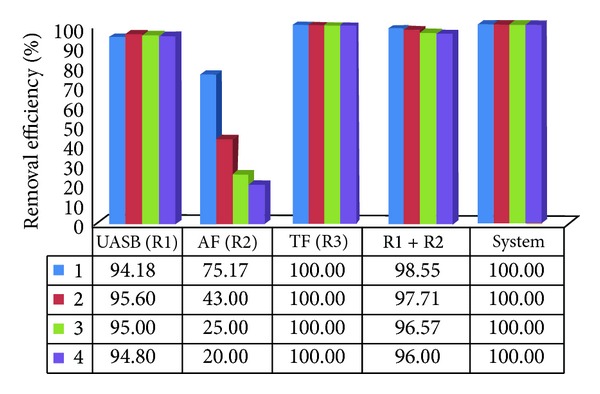
Average values of the removal efficiencies of* Ascari ssuum* eggs in the UASB reactors and anaerobic filter (R1 and R2) and R1 + R2 and system (R1 + R2 + R3 + Dec.) of treatment system II in assays 1, 2, 3, and 4.

Table 7 presents the results of the average numbers of* Ascaris suum* eggs obtained in the samples of inflow, effluents of the UASB reactor, anaerobic filter, trickling filter, and decanter of treatment system II. In the inflow, the number of* Ascaris suum *eggs found varied between 2.3 and 3.6 eggs L^−1^, averaging 2.7 eggs L^−1^ overall for system II. In the UASB reactor and anaerobic filter, the average was 0.14 eggs L^−1^ and 0.07 eggs L^−1^, respectively. In the effluent of the trickling filter and decanter, no* Ascaris suum* eggs were found, showing high efficiency of that combination in the removal of these organisms, as shown in Figure 8.

Cavalcante [32], working with anaerobic filters treating domestic sewage, obtained 99% removal efficiency for helminth eggs, resulting in a final effluent with values lower than 1 egg L^−1^.

In the treatment of domestic sewage, Passamani et al. [33] obtained an efficiency of 87.5% in UASB reactor (effluent with 24 eggs L^−1^), whereas no helminth eggs were detected in the effluent from biological filter placed in series, therefore achieving 100% removal efficiency, similar to those obtained in this work.

The variation in the number of eggs found in UASB effluents compared to other authors is quite significant. Zerbini et al. [34] and Souza [35] presented, respectively, a total of 195 and 229.9 eggs L^−1^, values much higher than those obtained in this study. With regard to the mean values obtained, the observed results are within the range presented in works of Cavalcante [32] and Passamani [33], averaging 0.65 and zero helminth eggs L^−1^ respectively.

In the anaerobic reactors of treatment systems I and II, the highest helminth removal efficiencies occurred with higher HRT in assays 1, 2, and 3. With the marked decrease in HRT in assay 4, the lowest helminth removals were caused principally by the reduction in the UASB reactor (R2) and in the AF of treatment systems I and II, in which HRTs were 4.5 and 5.2 h, respectively, as shown in Figures 7 and 8.

Leopoldino et al. [36], working with anaerobic filters to treat domestic sewage, observed a final effluent with means below 1 egg L^−1^, with 83.3% removal of helminth eggs. Egg size and density favour the removal by physical processes such as filtration and sedimentation, which are phenomena observed in anaerobic filters.

The obtained efficiency results of 99.11% and 100% for treatment systems I and II, respectively, were considered excellent in* Ascaris suum* eggs removal, meeting the health recommendations of the WHO for unrestricted use of the effluent for irrigation.

### 3.4. Determination of Helminth Eggs in Sludge

A large number of* Ascaris suum *eggs were found in the sludge blanket in the UASB reactors from treatment systems I and II a like 0.94 to 7.55 g^−1^ TS; in the bed, the egg count was higher, reaching values of 1.74 to 9.55 g^−1^ TS.

Of the analyzed samples, the highest concentrations were detected in the bed of the UASB in all assays and in the interstitial sludge from fixed bed of AF in assays 1 and 2.

The results obtained both in treatment system I and in treatment system II were above 0.25 egg g^−1^ TS in all assays, and consequently the sludge must be classified as type B according to CONAMA 375 [10], for which the threshold must be below 10 eggs g^−1^ TS and use is restricted, as shown in Table 8.

This study indicates that there is a tendency for eggs to be retained or settle onto the bed of the UASB reactor and be retained by adsorption in the biofilm formed in the support medium and the interstitial sludge from fixed bed of the AF and TF reactors, as they were found in low amounts or were not detected in the samples from the effluents.

In biological sludge we can find several different pathogenic microorganisms; however, the mere presence of an infectious agent in the sludge used in agriculture does not necessarily imply immediate transmission of diseases; it only characterizes a potential risk [37].

The real risk of infection for any individual actually depends on the combination of a series of factors, including resistance by pathogenic organisms against wastewater treatment and environmental conditions, infectious dose, pathogenicity, susceptibility and degree of immunity of the host, and degree of human exposure to outbreaks. Thus, in order for a microorganism present in an effluent used in agriculture to cause disease, it has to resist against the treatment processes employed and survive against the environment in sufficient numbers to infect a susceptible individual [37].

The results obtained in this research are of great importance for health and environmental engineering, as they evidence the importance of combined systems (anaerobic and aerobic), as a technology is capable of having a significant beneficial impact on publish health, by removing helminth eggs.

## 4. Conclusions

The removal of helminth eggs by the treatment systems was excellent, with 99.11% removal efficiency for system I and 100% for system II, showing values below 1 egg L^−1^ and meeting the health recommendations set by the WHO for unrestricted use of effluent in irrigation.

Coliform counts in the effluents of the treatment systems revealed a high potential of coliform removal, reaching percentage values of 99.51% for system I and 99,91% for system II, which resulted in final effluents with concentrations in the range of 1.00*E* + 04 to 1.00*E* + 07 MPN (100 mL)^−1^. The high concentrations of this parameter make the agricultural reuse possible only for restricted irrigation (grains, industrial crops, forage species, pastures, and trees).

When evaluated separately, the reactors showed lower removal efficiency for coliforms and helminth eggs than when analyzed as a system of reactors placed in series, evidencing the advantage of anaerobic reactor placed in series and the combination of anaerobic and aerobic reactors.

## Figures and Tables

**Table 1:** Operating time, average air temperature, hydraulic retention time (HRT), and volumetric organic loading (VOL), applied on the UASB reactors (R1 and R2) of treatment system I and in the UASB reactor (R1), anaerobic filter (AF), trickling filter (TF), and decanter of anaerobic treatment system II, in assays 1 to 4.

Attribute	Treatment system	Assays			
		1	2	3	4
Operating time (*d*)		112	69	100	130

Period (month/year)		06 to 10/11	10 to 12/11	01 to 04/12	05 to 09/12

average air temperature (°C)		22.1	22.9	23.3	19

	I				
HRT (h)	R1	40.0	30.0	20.0	11.0
	R2	16.3	12.2	8.1	4.5
	II				
	UASB	40.0	30.0	20.0	11.0
	AF	20.9	15.7	10.4	5.7
	TF	27.4	20.6	13.7	7.5
	D	20.0	15.0	10.0	5.5

	I				
VOL (g total COD (L d)^−1^)	R1	6.8	4.3	12.8	13.7
	R2	6.5	4.2	6.6	8.4
	II				
	UASB	5.6	5.2	9.7	18.7
	AF	1.4	2.1	6.0	7.8
	TF	0.65	1.2	1.2	5.3
	D	1.1	2.0	1.6	9.8

**Table 2:** Average values of the most probable number of total and thermotolerant coliforms in the inflow and effluents of UASB reactors (R1 and R2) placed in series of treatment system I, in assays 1, 2, 3, and 4.

Sampling site	Assay 1	Assay 2	Assay 3	Assay 4
	Total coliforms MPN (100 mL)^−1^			
Inflow	4.30*E* + 07	4.40*E* + 07	4.40*E* + 07	2.40*E* + 08
UASB R1	2.90*E* + 06	4.40*E* + 06	4.60*E* + 06	2.10*E* + 07
UASB R2	2.10*E* + 05	2.60*E* + 05	3.00*E* + 05	1.70*E* + 07

	Thermotolerant coliforms MPN (100 mL)^−1^			
Inflow	4.30*E* + 07	4.30*E* + 07	4.00*E* + 07	2.10*E* + 08
UASB R1	2.90*E* + 06	3.30*E* + 06	1.50*E* + 06	1.70*E* + 07
UASB R2	1.90*E* + 05	2.25*E* + 05	2.40*E* + 05	1.20*E* + 07

**Table 3:** Average values of the most probable number of total and thermotolerant coliforms in the inflow and effluents of treatment system II consisting of UASB reactor, anaerobic upflow filter (AF), trickling filter (TF), and decanter (D) placed in series, in assays 1, 2, 3 and 4.

Sampling site	Assay 1	Assay 2	Assay 3	Assay 4
	Total coliforms MPN (100 mL)^−1^			
Inflow	2.90*E* + 07	4.25*E* + 07	1.45*E* + 07	2.40*E* + 08
UASB	7.50*E* + 06	6.80*E* + 06	4.40*E* + 06	4.00*E* + 07
AF	2.30*E* + 06	4.40*E* + 05	6.20*E* + 05	2.00*E* + 07
TF	9.00*E* + 04	6.80*E* + 04	3.70*E* + 04	2.20*E* + 06
Decanter	2.40*E* + 04	4.60*E* + 04	3.00*E* + 04	1.20*E* + 06

	Thermotolerant coliforms MPN (100 mL)^−1^			
Inflow	2.90*E* + 07	4.05*E* + 07	1.40*E* + 07	2.20*E* + 08
UASB	6.40*E* + 06	5.40*E* + 06	2.20*E* + 06	3.70*E* + 07
AF	1.40*E* + 06	4.30*E* + 05	4.60*E* + 05	1.70*E* + 07
TF	2.00*E* + 04	4.85*E* + 04	3.40*E* + 04	1.50*E* + 06
Decanter	2.00*E* + 04	4.00*E* + 04	2.90*E* + 04	1.20*E* + 06

**Table 4:** Average of the most probable number (MPN g^−1^ TS) of total and thermotolerant coliforms in the sludge of the UASB reactors placed in series, at pilot scale of treatment system I, in assays 1, 2, 3, and 4.

Sampling site	Distance from entrance (mm)	Assay 1	Assay 2	Assay 3	Assay 4
		Total coliforms (MPN g^−1^ TS)			
UASB (R1)	400	1.50*E* + 08	6.50*E* + 08	4.85*E* + 08	9.10*E* + 08
	800	1.90*E* + 07	1.65*E* + 07	2.70*E* + 08	9.02*E* + 07
	1200	7.20*E* + 06	8.60*E* + 06	2.45*E* + 06	7.20*E* + 07
	1600	5.20*E* + 06	4.50*E* + 06	2.20*E* + 06	8.75*E* + 06

UASB (R2)	400	1.20*E* + 06	8.75*E* + 06	4.75*E* + 05	9.35*E* + 06
	800	9.30*E* + 05	1.06*E* + 06	4.00*E* + 05	5.30*E* + 05
	1200	9.10*E* + 05	4.95*E* + 05	2.90*E* + 05	4.10*E* + 05
	1600	6.00*E* + 05	2.60*E* + 05	3.60*E* + 05	3.60*E* + 05

		Thermotolerant coliforms (MPN g^−1^ TS)			
UASB (R1)	400	1.20*E* + 08	6.10*E* + 08	4.05*E* + 08	1.10*E* + 08
	800	1.90*E* + 07	1.50*E* + 07	2.30*E* + 08	8.20*E* + 07
	1200	0.72*E* + 07	8.60*E* + 06	2.25*E* + 06	6.30*E* + 07
	1600	0.72*E* + 07	4.50*E* + 06	2.00*E* + 06	7.80*E* + 06

UASB (R2)	400	1.00*E* + 06	2.00*E* + 06	4.15*E* + 05	6.50*E* + 06
	800	9.30*E* + 05	1.01*E* + 06	3.15*E* + 05	4.30*E* + 05
	1200	7.00*E* + 05	4.00*E* + 05	2.45*E* + 05	4.10*E* + 05
	1600	5.30*E* + 05	2.40*E* + 05	2.30*E* + 05	2.70*E* + 05

**Table 5:** Average of the most probable number (MPN/g^−1 ^TS) of total and thermotolerant coliforms in the sludge of the UASB reactor and upflow anaerobic filter (AF) placed in series, at pilot scale of the treatment system II, in assays 1, 2, 3 and 4.

Sampling site	Distance from entrance (mm)	Assay 1	Assay 2	Assay 3	Assay 4
		Total coliforms (MPN g^−1^ TS)			
UASB	000	8.40*E* + 08	7.05*E* + 09	6.05*E* + 09	9.50*E* + 09
	400	5.50*E* + 08	6.45*E* + 08	5.30*E* + 08	9.35*E* + 08
	790	3.70*E* + 07	6.00*E* + 08	3.75*E* + 08	9.05*E* + 08
	1180	3.00*E* + 07	5.40*E* + 07	3.70*E* + 08	5.00*E* + 08
	1570	2.40*E* + 07	3.10*E* + 07	3.00*E* + 07	4.00*E* + 07
AF	000	2.00*E* + 07	2.95*E* + 07	2.80*E* + 07	3.90*E* + 07
	380	1.90*E* + 07	2.45*E* + 07	2.00*E* + 07	3.30*E* + 07
	660	1.60*E* + 07	2.30*E* + 07	2.00*E* + 07	2.75*E* + 07
	940	1.40*E* + 07	1.95*E* + 07	1.85*E* + 07	2.07*E* + 07
	1220	1.20*E* + 07	1.50*E* + 07	1.35*E* + 07	2.00*E* + 07

		Thermotolerant coliforms (MPN g^−1^ TS)			
UASB	000	8.40*E* + 07	6.40*E* + 09	5.00*E* + 09	9.40*E* + 09
	380	5.30*E* + 07	6.01*E* + 08	4.02*E* + 08	8.75*E* + 08
	660	3.30*E* + 07	5.40*E* + 08	2.00*E* + 08	8.00*E* + 08
	940	2.90*E* + 07	3.70*E* + 07	1.80*E* + 08	4.35*E* + 08
	1220	2.00*E* + 07	3.00*E* + 07	2.52*E* + 07	3.95*E* + 07
AF	000	1.90*E* + 07	2.00*E* + 07	2.05*E* + 07	3.65*E* + 07
	380	1.70*E* + 07	1.95*E* + 07	2.00*E* + 07	3.07*E* + 07
	660	1.30*E* + 07	1.95*E* + 07	1.80*E* + 07	2.22*E* + 07
	940	1.20*E* + 07	1.01*E* + 07	1.77*E* + 07	2.00*E* + 07
	1220	1.05*E* + 07	0.90*E* + 07	1.09*E* + 07	1.90*E* + 07

**Table 6:** Results of the average numbers of *Ascaris  suum* eggs obtained in the samples of crude inflows and effluents of the UASB reactors (R1) and (R2) placed in series, at pilot scale, of treatment system I, in assays 1, 2, 3 and 4.

Assay	Inflow (egg L^−1^)	R1 (egg L^−1^)	R2 (egg L^−1^)
1	3.000	0.107	0.027
2	2.666	0.106	0.053
3	2.333	0.133	0.053
4	3.000	0.187	0.107

**Table 7:** Results of the average numbers of *Ascaris suum *eggs obtained in samples of crude inflows, effluents of the UASB reactor, upflow anaerobic filter, trickling filter, and decanter placed in series, at pilot scale, of treatment system II, in assays 1, 2, 3 and 4.

Assay	Inflow (egg L^−1^)	UASB (egg L^−1^)	AF (egg L^−1^)	TF (egg L^−1^)	Decanter (egg L^−1^)
1	3.667	0.213	0.053	0.00	0.00
2	2.332	0.107	0.053	0.00	0.00
3	2.333	0.107	0.080	0.00	0.00
4	2.667	0.133	0.107	0.00	0.00

**Table 8:** Results of the average numbers of *Ascaris suum *eggs g^−1^ TS in the sludge of UASB reactors placed in series, at pilot scale, of treatment system I and of the UASB reactor, upflow anaerobic filter (AF) placed in series, at pilot scale, of treatment system II, in assays 1, 2, 3, and 4.

Sampling site	Distance from the entrance (mm)	*Ascaris suum* eggs g^−1^ TS			
System I		Assay 1	Assay 2	Assay 3	Assay 4
UASB (R1)	400	9.27	9.03	8.22	5.59
	1200	5.23	5.36	7.55	4.94
UASB (R2)	400	1.74	2.19	6.34	3.74
	1200	0.94	1.58	4.83	3.03

System II					
UASB	200	9.55	8.60	7.04	3.30
	790	4.15	4.35	4.26	2.35
AF	200	4.07	2.41	1.99	1.90
	660	1.68	0.86	0.89	1.56
